# Infectious Entry Pathway of Enterovirus B Species

**DOI:** 10.3390/v7122945

**Published:** 2015-12-07

**Authors:** Varpu Marjomäki, Paula Turkki, Moona Huttunen

**Affiliations:** 1 Nanoscience Center, Department of Biological and Environmental Science, University of Jyväskylä, Jyväskylä 40014, Finland; paula.turkki@jyu.fi; 2 MRC-Laboratory for Molecular Cell Biology, University College London, London WC1E 6BT, UK; m.huttunen@ucl.ac.uk

**Keywords:** entry, echovirus, coxsackievirus A9, coxsackievirus B3, signaling

## Abstract

Enterovirus B species (EV-B) are responsible for a vast number of mild and serious acute infections. They are also suspected of remaining in the body, where they cause persistent infections contributing to chronic diseases such as type I diabetes. Recent studies of the infectious entry pathway of these viruses revealed remarkable similarities, including non-clathrin entry of large endosomes originating from the plasma membrane invaginations. Many cellular factors regulating the efficient entry have recently been associated with macropinocytic uptake, such as Rac1, serine/threonine p21-activated kinase (Pak1), actin, Na/H exchanger, phospholipace C (PLC) and protein kinase Cα (PKCα). Another characteristic feature is the entry of these viruses to neutral endosomes, independence of endosomal acidification and low association with acidic lysosomes. The biogenesis of neutral multivesicular bodies is crucial for their infection, at least for echovirus 1 (E1) and coxsackievirus A9 (CVA9). These pathways are triggered by the virus binding to their receptors on the plasma membrane, and they are not efficiently recycled like other cellular pathways used by circulating receptors. Therefore, the best “markers” of these pathways may be the viruses and often their receptors. A deeper understanding of this pathway and associated endosomes is crucial in elucidating the mechanisms of enterovirus uncoating and genome release from the endosomes to start efficient replication.

## 1. Species B Enteroviruses

**Figure 1:**
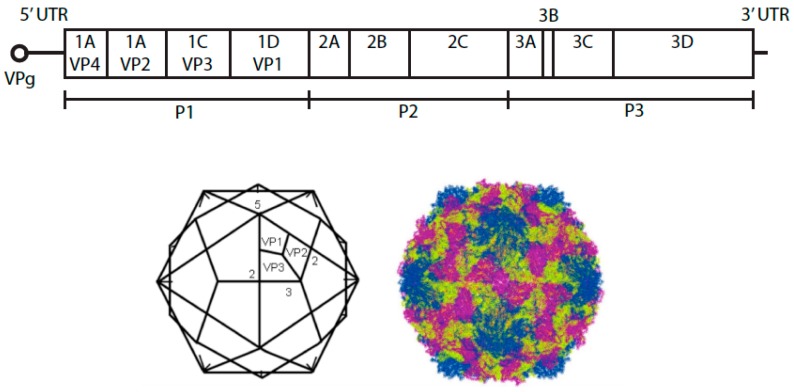
Schematic representation of the genome of an enterovirus B species. Below is the representation of the icosahedron structure of E1 showing the locations of two-, three-, and five-fold axes of symmetry and VP1 to VP3. The structure of E1 on the right was created using Jmol version 12.0.41 (an open-source Java viewer for chemical structures in 3D.) and the atomic coordinates downloaded from the Protein Data Bank, Brookhaven National Laboratory.

Species B enteroviruses (EV-B) consist of coxsackieviruses B1–B6 (CVB1–6), coxsackievirus A9 (CVA9), over 30 serotypes of echoviruses and more than 20 EV-B serotypes. They all share the same overall structure found in picornaviruses, a single stranded RNA genome and a roughly 7500 bp-long genome with a small genome linked virus-encoded protein (VPg) in the 5´end (Figure 1). The long 5´ untranslated region (UTR) is involved in the initiation of protein synthesis and the 3´UTR is involved in the synthesis of the negative strand RNA. The precursor protein P1 encodes four structural proteins VP1–VP4, which form 60 protomers and 12 pentagon-shaped pentamers (Figure 1). The precursor proteins P2 and P3 encode for seven non-structural proteins. Many of the intermediate cleavage products are functional proteins as well. Capsid proteins VP1–VP3 are found on the surface of the capsid, whereas VP4 is an internal capsid protein. In the icosahedral structure, beneath VP1, these viruses contain a hydrophobic pocket, which houses an aliphatic fatty acid. This structure is common to all enteroviruses, and it is linked to the stability of these viruses. Upon uncoating, the pocket factor is released from the virus particle. This is thought to result in increased destabilization and facilitates the release of the genome from one of the two-fold axis sites of the virus particle.

All EV-Bs can cause mild diseases with symptoms generally typical of those seen with cold. However, all have the potential to cause more serious acute and chronic infections. CVB serotypes can cause serious inflammations in secondary tissues, such as the myocardium, brain and pancreas leading to for example atherosclerosis and myocardial infarction [1]. There is evidence that many EV-Bs can contribute to the onset of type I diabetes [2,3]. Some CVA serotypes cause hand-foot-and-mouth disease. Enteric cytopathic human orphan viruses, (echoviruses), despite of being orphan viruses are known to cause various non-specific viral infections and nervous disorders such as meningitis. The primary infection pathway of these acid-stabile viruses is the fecal–oral route. As enterovirus receptors are typically widely available molecules, such as integrins, decay accelerating factor (DAF) and Coxsackie and adenovirus receptor (CAR), it is of no wonder that EV-Bs may cause such a variety of acute and chronic infections in various tissues.

The receptors responsible for binding and facilitating entry involve various types of cell surface molecules. However, recent results have shown that many members of these viruses share some crucial characteristics for their infectious entry to cells. In the following sections, we go through the present knowledge of the entry of E1, CVA9 and CVB3 and finally present the nature of the “EV-B entry pathway”.

### 1.1. Echovirus 1 (E1)

E1 is a prevalent enterovirus among children. In a recent study of more than 500 genetically susceptible children to type 1 diabetes, about 25% of all children had previously encountered E1 [2]. E1 binds selectively to the α2β1 integrin, a collagen-binding integrin, which is an abundant receptor on the cell surface of most cell types. The binding affinity of E1 to the α2β1 integrin is much greater than that of the rod-shaped collagen molecule to the integrin, thus helping E1 to compete with the binding [4]. Although simultaneous binding of E1 and collagen to the integrin is not possible due to overlapping binding sites, ample integrin usually seems to be available to facilitate virus infection even in a 3D collagen environment (unpublished observation). Another possible receptor candidate for E1 is β2-microglobulin, as monoclonal antibodies against β2-microglobulin can totally abolish cell binding *in vitro* [5]. However, thus far, only α2β1 integrin has been shown to be involved in effective cell entry, with cells lacking this integrin displaying very low infectivity. The potential importance of β2-microglobulin in binding and infection (e.g., in the tissue context) remains to be shown. Only human or monkey cell lines support infection as the integrin sequences in mouse cells differ too much from the human counterparts.

The entry of E1 would be expected to closely resemble the normal entry and recycling of the α2β1 integrin. However, this is not the case: E1 stimulates the uptake of a new pathway and new endosomal vesicles and does not much mix with the continuous endosomal entry and recycling [6]. Normally, the β1 integrins use early endosomes as sorting stations for either recycling to the plasma membrane, or by taking a longer pathway via perinuclear recycling endosomes [7]. Many of the players in these pathways are well known, and they are mostly shared by other recycling receptors using the clathrin dependent pathway. Several approaches used to monitor recycling showed conclusively that E1-induced α2β1 integrin-containing vesicles are not recycled back to the plasma membrane [6].

As research showed that α2β1 integrin mainly resided on the raft areas on the plasma membrane, both specialized caveoli as well as the planar raft areas, the first attention was caught by these special lipid domains [8]. Immunoisolation of infective viral particles with affinity purification using antibodies against caveolins suggested that early entry would occur through flask-shaped caveoli structures [5]. However, detailed colocalization studies showed that the association with caveolins occurred later with little contact in the very first minutes [9]. Studies of SV40 by Helenius´s group showed that entry through vesicular caveolae was possible but very slow, whereas entry via planar raft domains of the plasma membrane supported much more efficient entry [10]. In our previous study, E1 was occasionally found in caveolae, but they acted only as a minor route of entry. Direct uptake from raft domains, rich in glycosyl phosphatidyl inositol (GPI)-anchored proteins allowed efficient entry to spacious smooth-surfaced endosomes [5].

Due to the association of integrin with lipid domains, cholesterol was suspected to play a role in the E1 entry and infection [8]. Indeed, various perturbations on cholesterol homeostasis, such as sequestering the cholesterol by methyl β-cyclodextrin, aggregating cholesterol with filipin or reducing the biosynthesis of cholesterol by ketoconazole treatment, all arrested the uptake on the plasma membrane, confirming that cholesterol was necessary for the uptake [11]. Quantitative confocal microscopy showed that the internalized endosomes were rich in cholesterol, and that the perturbations of cholesterol totally arrested infection even in cytoplasmic endosomes.

As actin plays a crucial role in most membrane trafficking steps, it was no surprise that actin was somehow involved in the entry of E1 [9,12]. The various drugs perturbing the dynamics of actin showed that uptake was arrested on the plasma membrane. In addition, Rac1, an Rho GTPase, which regulates the dynamics of actin, was regulating also E1 entry, whereas other members of Rho GTPases had no apparent effect [9]. The serine/threonine p21-activated kinase Pak1, which is an effector of Rho GTPases such as Rac1, was activated early in the E1 uptake with the highest activation reached 30 min post infection [9]. Pak1 phosphorylates downstream the dynamin-like regulator of macropinocytic entry, C-terminal-binding protein-1/brefeldinA-ADP ribosylated substrate (CtBP1/BARS) [13]. This dual-functioning protein is an interesting molecule, which on the one hand reacts to cellular metabolites and mediates messages to gene transcription, whereas the CtBP1/BARS found close to the plasma membrane regulates closing of the macropinocytic cup. This was shown for E1 uptake as well as for EGFR uptake [13]. In the cell types, where dynamin is more prevalent, dynamin may take the same role as CtBP1/BARS of facilitating the pinching of the plasma membrane invagination even for E1 [12].

The host cell regulators of entry that act at the plasma membrane include phospholipase Cγ (PL Cγ) and Protein kinase Cα (PKCα) [8,9]. The activation of PLCγ leads to the production of inositol trisphophate and diacylglycerol, which in turn activates plasma membrane PKCα. The basal activation status of PKCα is usually high but becomes even higher after receptor clustering due to virus binding on the plasma membrane [8]. The uptake of E1 was negative for several rab modulators (rab5, rab 7, rab 31, rab21), that are known to mediate endocytic membrane traffic. Dominant-negative rab5 showed a moderate inhibition of entry, which seemed somewhat unexpected as functional markers of entry, such as transferrin and 3,3´-dioctadecylindocarbocyanine-low density lipoprotein (diL-LDL) did not cross the E1 pathway [6]. However, other examples of viruses that seemed to use macropinocytic uptake but were still regulated by some rab5 effectors or dominant negative rab5 were later reported. In particular, rabankyrin, the rab5 effector was shown to regulate entry through macropinocytosis [14].

Unfortunately, studies have yet to identify a definite marker of E1 entry. The failure to find such a marker is likely due to the fact that E1 pathway is triggered upon virus binding on the receptor. Thus, it does not take part in membrane trafficking, which would circulate other receptors along this pathway. All tested markers linked with the classical lysosomal entry pathway seemed to avoid this pathway. The markers of early endosomes, rab5, transferrin, and markers for the later structures, including internalized diL-LDL, lyso-bis-phosphatidic acid (LBPA), rab7, Lamp1 and CD63 were not associated with E1 endosomes. The only cointernalized molecules were fluid-phase markers such as dextran or horse radish peroxidase (HRP) that were simultaneously taken up from the extracellular milieu [9].

One of the earliest findings suggesting that endosomes along the E1 pathway were not acidified was obtained by cointernalized fluorescein isothiocyanate (FITC)-dextran that was effectively taken to the triggered endosomes with E1 [9]. Acid-sensitive FITC did not show any decrease of fluorescence even after 1 or 2 h of entry, whereas in control cells without E1 triggered endosomes, the fluorescence of FITC dextran was typically faded. Other clear indications of a neutral pathway were the lack of colocalization with low pH targeted lysotrackers and a lack of effect by bafilomycin A1, which effectively inhibits the proton pump ATPase, on virus infectivity [6]. In addition, nocodazole treatment had no effect clearly showing that although a microtubule-dependent transfer to the perinuclear region is typical of E1 entry, this perinuclear targeting and transfer to possible acidic endosomes was not needed for infection [12]. Final proof for the neutrality of the vesicles came from the intraendosomal real-time pH measurements, which were executed by the ratio of acid-sensitive and acid-insensitive secondary antibodies that were specifically targeted to the endosomes [15]. The pH seemed to reduce to just below pH 7, whereas epidermal growth factor receptor (EGFR) quickly ended up in endosomes with a pH lower than 6 [6]. The Na^+^/H^+^ exchanger seemed to be important for the entry because an amiloride analogue, 5-(N-Ethyl-N-Isopropyl) Amiloride (EIPA), effectively blocked the entry to early structures [9]. The consensus in the literature at the time was that EIPA arrested the entry on the plasma membrane. However, at least in the case of E1, the ruthenium red labeling of the cells during fixation clearly showed that the arrested structures containing the virus were not associated with to the plasma membrane [9]. Later, EIPA was shown to inhibit alkalinization of the cytoplasm during macropinocytic entry by perturbing the activation of Rac1 and remodeling actin linked to macropinocytosis [16]. The aforementioned findings suggested that local changes occurred in the proton and sodium concentration. However, whether these changes contribute to the ionic conditions that contribute to virus uncoating remains unclear.

A recent study of another echovirus also belonging to EV-Bs, echovirus 7 (E7) reported an interesting finding with respect to entry to early and late endosomes [17]. In that study, entry to acidic endosomes was not essential for virus uncoating. Furthermore, acidification or lysosomal enzyme activity was not needed for E7 infection. These results led the authors to suggest that E7 may travel only through classical endosomes but perhaps needs yet another structure for uncoating.

The endosomal structures accumulating E1 and its receptor α2β1 integrin, increased in size and grew intraluminal vesicles (ILVs) [9]. These multivesicular bodies (MVBs) were very similar to the late endosomes that had been well characterized earlier [18,19]. The biogenesis of intraluminal vesicles (ILVs) was previously shown to involve the action of ESCRT (endosomal sorting complex required for transport) proteins [20]. In the case of E1, Dominant-negative Vps4 also clearly perturbed both the biogenesis of MVBs as well as viral infection [15]. Other members of the ESCRT family, such as Hrs, Vps 37A and Vps 24 were shown to associate with viral MVBs suggesting that the ESCRT machinery produced the MVBs [15]. The integrins mediate binding of the viral capsid in the MVBs leading to virus capsids floating in the endosomal lumen. It is unclear whether the viral genome that is released from the viral capsid floats freely in the lumen or whether it is temporarily taken up inside the ILVs. Furthermore, it is unclear whether the high viral load used to study the entry of E1 have affects on the biogenesis of ILVs. A more detailed EM study using high-pressure fixed samples of the endosomal structures that accumulate E1 and its integrin receptor, showed enlargement of the MVBs and ILVs and appearance of openings in the limiting membrane of the MVBs and ILVs [21]. A biochemical approach suggested that the permeability of these structures increased after 2 h post infection (p.i.) [21]. Later time points also showed clear signs of degradation of the MVBs [6]. The most plausible explanation for viral genome egress is that the virus-derived VP4 molecules produce small pores in the endosomal limiting membrane thereby allowing the genome to be released to the cytoplasm [22]. However, the exact structural changes that contribute to the genome egress remain to be elucidated, as do the host cell factors that contribute to this event. Previous research demonstrated that the replication structures of enteroviruses included first single- and later double-membrane vesicles that were enwrapped by multiple cisternae [23]. A similar kind of membrane structure was observed in the cytoplasm of E1 infected cells (unpublished data). However, whether the MVBs are associated with early replication machinery remains to be studied.

While studying the fate of the E1 receptor α2β1 integrin in cells during viral infections we observed that, despite the block in recycling of the integrin back to the plasma membrane, integrin was gradually degraded in the infected cells. The neutral calpain proteases were activated during E1 infection, and these activated proteases were associated with virus-loaded endosomes in the cytoplasm [6]. The integrin C-terminus is known to act as a substrate for calpains, which could already attract calpains to the vesicles. However, the N-terminus of the integrin that faces the lumen of the vesicles was also degraded at later time points, and the N-terminus was degraded by calpains *in vitro* [6]. Calpains are ubiquitous molecules in the cytoplasm and need strict regulation to limit their activity. Due to their high number of actions, inhibiting their activity by drugs in the hope of studying detailed activities is impossible as drugs inhibit several simultaneous events in the cell. However, it was clear that calpain inhibition using the pan-inhibitor calpeptin, did not inhibit the entry of E1. Instead, it caused an accumulation of E1 in the cytoplasmic endosomes and efficiently blocked replication of the virus. It is possible that calpains contribute to the degradation of the MVBs and that they are required for an efficient start of the enterovirus replication in the cytoplasm.

### 1.2. Coxsackievirus A9 (CVA9)

Light and EM observations of the early CVA9 infection of cells suggested that, in common with E1, CVA9 infection strongly modulated actin on the plasma membrane and caused ruffling and large invaginations for the virus to gain entry [24]. Despite the possible use of members (e.g., αVβ6 and β3 integrins) of the αV integrin family as receptors, these did not cointernalize with the virus in endosomes [25]. Interestingly, β2-microglobulin was suggested to play a role in CVA9 infection [25]. suggesting that it may have a common role in enteroviral infection or for picornaviruses, and that its role may be linked to that of the heat shock 70 kDa protein 5 [26].

Similar to E1, the entry of CVA9 was shown not to be dependent on the clathrin pathway as illustrated by a study of dominant-negative constructs against clathrin adaptors Eps 15 or Ap180 and by drug treatment of chlorpromazine [25]. CVA9 was shown to first gain entry from lipid microdomains where the receptors are located, and its entry was sensitive to perturbations of the cholesterol content [26]. As with E1, the internalized CVA9 does not colocalize with clathrin endosome markers on the early endosomes or late endosomes/lysosomes [24]. A very similar inhibitory effect by the rab5 dominant negative construct underlines the possibility that the entry of CVA9 is closest to macropinocytosis [24]. In addition, CVA9 showed a very strong inhibitory phenotype upon treatment on EIPA further suggesting that macropinocytosis is indeed in use [24,25]. The EIPA induced perturbation of the local proton concentration compromised the action of Rac1 [16]. Rac1 regulation was also independently confirmed by the drug NSC23766 [24]. Interestingly, Rac1 still seemed to exert activity 1 to 2 h p.i. (*i.e.*, after the entry of the virus to cytoplasmic endosomes) suggesting that Rac1 regulated action has its effects on virus infection [24]. Similar to this, EIPA allowed E1 to enter to cytoplasmic early type endosomes after which the biogenesis of MVBs and infection were halted [9]. The role of of Rac1 or local ionic conditions in promoting viral infection remains to be studied.

The MVBs were shown to be important in CVA9 infection, as found with E1 [24]. Perturbation of the members of the ESCRT family by the siRNA method, overexpression of components and dominant negative constructs, all compromised viral infection. Similarly, MVBs were shown not to acidify during infection confirming that the neutral endosomes are used by EV-B members in general [24]. As CVA9 studies confirm that MVBs indeed are essential for enteroviral infection, it will be important to discover the structural or functional aspects of neutral MVBs that promote enteroviral infection.

### 1.3. Coxsackie B Viruses

There are six serotypes of CVB (CVB1–6). All CVB species can bind to and internalize via CAR [27,28], which is a transmembrane protein and a member of the tight junction (TJ) proteins [29]. Due to its location in TJ, in polarized tissues, viruses do not have free access to CAR. In addition to CAR, CVB1, CVB3 and CVB5 have also been shown to bind DAF [30,31,32]. DAF is a GPI-anchored membrane protein, which is abundantly expressed on the apical surface of cells where viruses have free access to it. DAF is not involved in internalization. However, it plays a role in signaling events and in the transport of the virus from the apical membrane to TJ and the CAR [32,33]. In addition to CAR and DAF, a PD variant CVB3 (CVB3-PD), was also shown to bind and internalize via heparan sulfates [34,35], and all CVB types have been shown to bind nucleolin [36]. Unlike E1 [5,11] and CVA9 [24], the conformational change of CVB and subsequent uncoating take place already during virus-receptor interactions. With CVB, the induction of the formation of intermediate particle requires only an association with CAR and not DAF [33]. Although the virus-receptor interaction can result in the formation of intermediate particles, the internalization of the viral capsid seems to be required for the release of the genome [37]. Internalization of CAR with the virus in the cells depends on the cell type [32,37,38].

The ability or inability of CVB to use DAF is most probably an adaptation to the conditions that the virus faces in its lifecycle. While crossing epithelial, endothelial or placental barriers, the virus depends on attachment receptor, such as DAF, to obtain access to CAR in TJ. Furthermore, CVB was shown to be able to adopt to bind to several other cell surface molecules in a receptor-limited environment involving group selection of minority variants [39,40] The use of additional attachment factors most probably allows the virus to survive until CAR becomes available again [41].

#### Entry of Coxsackie Virus B3

Although CVB species have been shown to adapt to use other cell surface molecules in addition to CAR and DAF, [34,35], CAR is the common entry receptor utilized by all CVB types. In polarized conditions where CAR is located in TJ and normally unavailable to the virus, the virus most often binds to DAF on the apical surface.

At the cell surface, DAF is commonly associated with lipid rafts. Therefore, not surprisingly, cholesterol-modifying agents such as filipin and methyl-β-cyclodextrin (MBCD) were shown to inhibit CVB3 infection [32,37]. The binding of the virus to DAF on the cell surface produces clusters of the receptor and leads to translocation of the virus-receptor complex to TJ. Clustering and translocation of the receptor can also be achieved without the virus by means of clustering antibodies, similar to what has been shown for E1 receptor α2β1 integrin [8,32]. The perturbation of cholesterol was shown to inhibit the transfer of clustered DAF from the apical side to TJ suggesting that lateral movement of DAF needs intact cholesterol domains [32]. The interaction between CVB3 and DAF was shown to activate tyrosine kinase Abl, which further triggered Rac1 activation. Various drug treatments affecting actin dynamics and Rac1 activation (e.g., EIPA treatment) arrested the translocation of CVB3 to TJ indicating that Rac1-mediated actin dynamics was involved in cell-surface translocation [32]. In addition to Rac1, the activation of RhoA and Cdc42 was induced during early entry suggesting that more complex actin dynamics were involved in the infective pathway of CVB3 in polarized conditions [32]. Furthermore, DAF-virus interactions were shown to trigger Fyn a member of the Src family of kinases, leading to phosphorylation of caveolin and accumulation of phosphorylated caveolin in the TJ, a requirement for subsequent entry steps. DAF-mediated activation of Abl, Src, and Fyn are early events in the viral entry, and can also be induced by antibody-mediated DAF clustering.

When CVB types are introduced to cells, TJs partially lose their barrier ability and become more permeable, suggesting that the virus causes structural changes in the junctions. Increased permeability of TJs can be observed 15 min p.i., and CVB3 was shown to be concentrated at TJ with CAR 20–30 min p.i. [42]. In polarized cells, CAR did not internalize with the virus in endosomes and another TJ protein, occludin, was internalized instead. Occludin and the virus entered through same entry pathway and entry of both was dependent on caveolin phosphorylation and macropinosome regulators Rab34, Ras and Rab5. EIPA and rottlerin inhibited internalization, further demonstrating the Na^+^/H^+^ exchanger and PKC regulated the macropinocytotic entry. Both occludin and the virus located in macropinocytic vesicles. However, interestingly, they remained separate from each other. The exact role of occludin in CVB3 infection is not known, but it is essential for the entry of the virus [32,42].

Once DAF has delivered the virus to CAR in the TJs, the virus-CAR interaction leads to the formation of the intermediate particle [32,42]. After 1 h p.i., the virus can be seen in vesicles in the cytoplasm that contain caveolin but not clathrin or any of the classical endosomal markers. This again is very similar to E1, showing colocalization with caveolin-1 but not with any of the known classical endosomal markers [9]. The uptake of CVB3 was shown to be unaffected by mutants of Rab7, but it was inhibited by dominant negative and constitutively active Rab5 [32,42], similarly to what was observed with E1 [7]. Neither dynamin nor the clathrin regulator Eps15 was involved in the internalization. Although intermediate particle formation took place during receptor interactions, VP4 was internalized in the cells with the capsid, suggesting that it might play a role later in the virus lifecycle [32]. VP4 is thought to be absent from the intermediate particle (also called as an A-particle), leading to the formation of the 135S particle. The presence of VP4 in CVB3 intermediate particle point to the presence of modified particle form that is between 135S and 160S. This is very similar to what we have observed in previous studies of E1: Upon receptor binding, in sucrose gradient the E1 particle was somewhat lighter than 160S, but it retained its VP4 to a large extent during entry to cytoplasmic endosomes [5]; and our recent unpublished results).

Pretreatment of cells with genistein or protein phosphatase 2 (PP2) inhibited CVB3 infection, demonstrating that tyrosine kinases and phosphatases were involved in the entry of CVB3 entry [32,43]. Furthermore, the PP2 treatment trapped the viruses at the cell surface at an intermediate state with their entry suspended. Neither DAF nor CAR could be visualized with intracellular virus suggesting that the receptors are not internalized with the virus [33]. New viral RNA started emerging at around 4 h p.i. in epithelial cells, which was close to what was also observed for E1 and CVA9 [24,32,44].

In polarized endothelial cells, the signaling involved in the entry of the virus is different to that of epithelial cells described above [32,42,43]. Similar to epithelial cells, DAF mediates the actin-mediated cell surface transport and hands virus over to CAR in the TJs. Although the entry depended on caveolin and dynamin, it did not require clathrin. Furthermore, in endothelial cells the entry was dependent on the release of Ca^2+^ and the subsequent activity of calpains. The DAF-CVB interaction induced the activity of the Src family of kinases and PLCγ, resulting in the immediate release and quick depletion of intracellular Ca^2+^ stores leading to calpain activation. When calpain activity was inhibited, viruses were arrested for extended periods in large (>500 nm) vesicles which were devoid of endosomal markers but heavily associated with calpain-2 and the caveolae marker cholera toxin B [43]. The calpain activity and its association with endosomal vesicles was very similar to the events that occurred during entry of E1 [6]. This suggests that calpains may have a more general role in the outcome of enterovirus infection.

In nonpolarized HeLa cells where both CAR and DAF are on the apical surface and freely available to viruses, DAF binding does not play such a major role in viral entry. Both DAF-binding CVB3-RD and nonbinding CVB3-Nancy can readily infect nonpolarized HeLa cells, and their internalization seems to rely on the same pathway [37]. However, DAF bound CVB was more abundant on the cell surface because DAF offers more binding possibilities than CAR [37]. Thus, although DAF-mediated signaling is not required in the entry of CVB3, DAF enhances binding and internalization, giving these viruses an advantage in terms of entry to the cells. Furthermore, in contrast to polarized cells, in nonpolarized Hela cells, CAR and CVB can be found in the same intracellular vesicles [37].

The prevention of endosomal acidification with the lysomotropic agent NH_4_Cl and bafilomycin A treatments had no effect on virus infection, showing that endosomal acidification was not needed for CAR-mediated CVB3 infection [37]. Similar observations were reported for E1 and CVA9 [6,24]. Information is lacking on the nature of endosomal vesicles where CVB3 accumulates (*i.e.*, whether they are multivesicular structures, as observed with E1 and CVA9).This information will be important in evaluating the late endosomal events leading to successful replication by enteroviruses.

## 2. EV-B Infectious Entry Pathway

**Figure 2:**
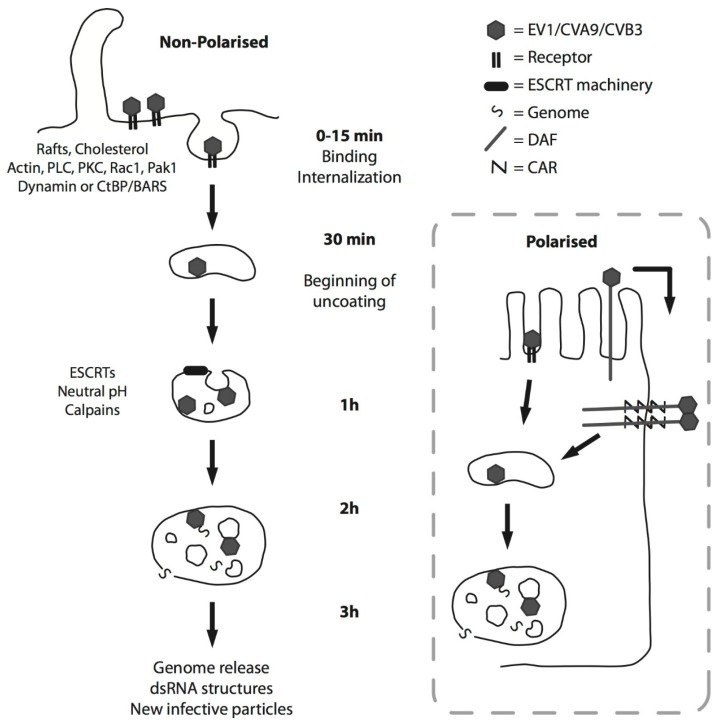
A schematic representation of the EV-B entry pathway in both nonpolarized (**left**) and polarized cells (**right**). See Section 2 for details.

The data acquired so far on the different EV-B species members point to a presence of dedicated EV-B infection pathway (Table 1 and Table 2). This pathway is a triggered pathway, starting from the raft domains on the plasma membrane (Figure 2). Virus binding to receptor on the plasma membrane causes clustering of the receptors, which triggers the signaling events leading to internalization of the virus in cytoplasmic endosomes. The first host cell molecules that are usually involved in viral entry are cholesterol and actin. The activation of PLC leads to production of inositol phosphate and diacylglycerol. These in turn, activate PKCα, which aids the entry of the virus. Rac1 is then activated, leading to actin remodeling and downstream Pak1 and CtBP/BARS phosphorylation. CtBP/BARS or dynamin (depending on the cell type) facilitates the pinching of clathrin-independent carriers from the plasma membrane. After 15–30 min, the virus is found in endosomes in the cytoplasm. These endosomes do not have acidic environment, although this macropinocytic entry is dependent on the action of Na^+^/H^+^ exchanger. The activity of these antiporters possibly leads to local changes in Na^+^ and H^+^ concentrations. These events have been shown to be crucial for Rac1 activation, and the inhibition of the Na^+^/H^+^ exchanger was shown to interrupt internalization in the early stages. With regard to E1 and CVA9, the structures develop into ESCRT-driven MVBs, which have neutral environment. Thus, they do not mix to any great extent with lysosomal structures, which have an acidic pH. It will be interesting to learn whether the infection of CVB species also relies on the biogenesis of MVBs.

**Table 1:** Different treatments and their effect on E1, CVA9 and CVB3 infection.

Target	Treatment	E1	CVA9	CVB3	References
Clathrin	Chlorpromazine	−	−	+/−	[25,35,37,38,45]
	Ap180	−	−		[12,25]
	DN Eps15	+/−	−	−	[12,25,28,32,44,45]
	siClathrinHeavyChain	+/−		−	[45]
	Clathrin RNAi			−	[37,38]
Dynamin/CtBP/BARS	Dynasore	+/−	+	+/−	[9,25,37,38,44,45]
	DN Dynamin	+/−	+	+/−	[9,12,25,37,38,44,45]
	siDynamin	+		+/−	[37,38,44,45]
	CtBP/BARS siRNA	+			[15,45]
Caveolin	siCaveolin	−		+/−	[37,38,45]
	DN Caveolin	+/−	−	+/−	[9,12,25,32,37,44,45]
Cholesterol	MβCD	+	−	+	[5,12,25,32,37,38,45]
	Progesterone+Nystatin	+	−		[12,25]
	Filipin	+/−		+/−	[11,37,38,45]
	Nystatin	+		−	[11,35,38]
	Ketokonazole	+			[11]
	U18666A	−			[11]
	Cholesterol oxidase			+	[38]
Actin	Cytochalasin D	+/−	−	+/−	[8,9,12,25,32,38,45]
	Latrunculin A	−	−	+/−	[12,25,32,38]
	Jasplakinolide	+/−	+	+	[8,9,12,25,45]
Rac1	NSC23766		+	+	[24,32]
	DN Rac1	+		+	[9,32,45]
	siRac1	+			[9]
Pak1	IPA−3	+	−	+	[24,45]
	DN Pak1	+			[9]
PLC	U−73122	+	+	+/−	[9,24,44]
PKC	Bisindolylmaleimide	+			[8,12]
	Safingol	+			[8,12]
	PMA	+			[8]
	Rottlerin	+		+	[38,42,45]
	DN PKCa	+			[9]
Acidification	Bafilomycin A1	−	−	+/−	[24,35,37,45]
	NH_4_Cl		−	−	[25,37]
Microtubules	Nocodazole	−	−	+	[12,24,25,38]
Na^+^/H^+^ exchanger	EIPA	+	+	+	[9,24,25,38,42,45]
ESCRT	Hrs	+	+		[15,24]
	siTSG101	+/−			[15]
	siVps37A	+			[15]
	siVps24	+			[15]
	DN Vps4	+	+		[15,24]
PI3K	LY290042	+/−	−		[9,24]
	Wortmannin		−	−	[24,25,38]
Calpain	Calpeptin	+		+	[43,44]
	Inhibitor1/2	+			[44]
	siCalpain(1/2)	+		+/−	[43,44]
	ALLN			+	[43]
	Inhibitor III			+	[43]
Tyrosine kinase	Genistein	+		+	[12,32,37,38,43]

+ means that the drug or treatment had an effect on the life-cycle of the virus; − means there was no effect; +/− refers to a minor effect or mixed results from various studies.

**Table 2:** Colocalization of endosomal structures or proteins associated with E1, CVA9 and CVB3.

Structure	Marker	E1	CVA9	CVB3	References
Clathrin	Clathrin			−	[32]
Caveolae	Caveolin	+	−	+	[5,8,12,25,32,43]
Cholera Toxin B	Cholera Toxin B	+/−		+	[9,32,43]
Macropinosome	Dextran	+	−	+	[9,25,44,45]
Early endosome	EEA1	−/+	−		[8,9,15,24,45]
	Rab5			+/−	[42,43]
Recycling endosome	Transferrin	−			[5]
Late endosome	CI−MPR	−		(−^1^)	[5,9,32]
	LBPA	−			[15]
	Rab7	−	−		[15,24]
Late endosome/	CD63	−		(−^1^)	[9,15,32]
Lysosome	Lamp1	−	−		[15,24]
	Lamp2	+			[45]
	LysoTracker	−	−		[12,24]
	Dil−LDL	−	−		[15,24]
Calpain	Calpain	+		+	[6,43]

^1^ CVB3 does not colocalize with endosomal markers (data not shown) [33]; + means colocalization of the virus with the marker; − means no colocalization; +/− refers to mixed results.

Following the successful entry of the virus to cytoplasmic endosomes, uncoating continues for at least 2 h. The genome is then released into the cytoplasm, where viral replication commences. Calpains have been indicated in the infectious pathway of these enteroviruses. Calpains are activated during viral entry, and they associate with endosomal structures. Calpains have diverse roles in cells, including cytoskeletal modeling, trafficking, degradation, and cell death. More information is needed to unravel their exact functions during enteroviral infection. It remains to be elucidated whether the characteristics of the EV-B entry pathway are shared by other members of picornavirus family.
